# In Vitro Anti-Inflammatory Effects of *Symplocos sumuntia* Buch.-Ham. Ex D. Don Extract via Blockage of the NF-κB/JNK Signaling Pathways in LPS-Activated Microglial Cells

**DOI:** 10.3390/plants11223095

**Published:** 2022-11-14

**Authors:** Jae Sung Lim, Jaehoon Bae, Seoyoung Lee, Da Young Lee, Lulu Yao, Namki Cho, Tran The Bach, Narae Yun, Su-Jin Park, Young-Chang Cho

**Affiliations:** 1 College of Pharmacy and Research Institute of Pharmaceutical Sciences, Chonnam National University, 77 Yongbong-ro, Gwangju 61186, Republic of Korea; 2 Functional Biomaterial Research Center, Korea Research Institute of Bioscience and Biotechnology, 181 Ipsin-gil, Jeongeup-si, Jeonbuk 56212, Republic of Korea; 3 Department of Research, Lab Technology System Co., Ltd., Daejeon 35365, Republic of Korea; 4 Institute of Ecology and Biological Resources, Vietnam Academy of Science and Technology (VAST), 18 Hoang Quoc Viet, Cau Giay, Ha Noi 122000, Vietnam; 5 International Biological Material Research Center, Korea Research Institute of Bioscience & Biotechnology, 125, Gwahak-ro, Yuseong-gu, Daejeon 34141, Republic of Korea

**Keywords:** *Symplocos sumuntia* Buch.-Ham. ex D. Don, anti-inflammatory activity, microglial cells, MAPK signaling pathways, NF-κB translocation

## Abstract

*Symplocos sumuntia* Buch.-Ham. ex D. Don (*S. sumuntia*) is a traditional medicinal herb used in Asia to treat various pathologies, including cough, stomachache, tonsillitis, hypertension, and hyperlipidemia. Although the anti-inflammatory activity of *S. sumuntia* has been reported, little is known about its anti-inflammatory activity and molecular mechanisms in microglial cells. Therefore, we investigated the inhibitory effects of *S. sumuntia* methanol extract (SSME) on the inflammatory responses in lipopolysaccharide (LPS)-treated BV2 cells. The SSME significantly inhibited the LPS-stimulated inducible nitric oxide synthase and cyclooxygenase-2 expression, as well as the production of nitric oxide (NO), a proinflammatory mediator. The production of proinflammatory cytokines, including interleukin (IL)-6, tumor necrosis factor-α, and IL-1β, was suppressed by the SSME in the LPS-induced BV2 cells. The mechanism underlying the anti-inflammatory effects of SSME involves the suppression of the LPS-stimulated phosphorylation of mitogen-activated protein kinases (MAPKs) such as JNK. Moreover, we showed that the LPS-stimulated nuclear translocation of the nuclear factor-κB (NF-κB)/p65 protein, followed by IκB degradation, was decreased by the SSME treatment. Collectively, these results showed that the SSME induced anti-inflammatory effects via the suppression of the MAPK signaling pathways, accompanied by changes in the NF-κB translocation into the nucleus. Therefore, SSME may be employed as a potential therapeutic candidate for various inflammatory diseases.

## 1. Introduction

Inflammation is a complex biological process of the immune system that is commonly involved in the pathogenesis of many chronic diseases, including cancers and cardiovascular and metabolic syndrome [1,2,3,4]. Inflammation mediated by microglia, the resident innate immune cells in the central nervous system (CNS), may play an important role in Parkinson’s disease (PD), Alzheimer’s disease (AD), multiple sclerosis (MS), amyotrophic lateral sclerosis, stroke, and traumatic brain injury [5]. In a proinflammatory state, microglial cells produce and release reactive oxygen and nitrogen species (ROS and RNS) and cytokines, such as tumor necrosis factor-α (TNF-α) and interleukin (IL)-1β or IL-12 [6]. Inducible nitric oxide synthase (iNOS) is an enzyme that generates nitric oxide (NO) from ʟ-arginine [7,8]. NO, derived from iNOS, plays a key role in various physiological conditions and the regulation of immune responses. Members of the mitogen-activated protein kinase (MAPKs) family are involved in proinflammatory gene expression during the inflammation process [9,10]. Developing therapeutic compounds that attenuate proinflammatory cytokines and inflammatory mediators in the microglial cells may be useful in treating neurodegenerative diseases such as AD, PD, and MS [11,12].

*Symplocos* is a genus of flowering plants with many species distributed in Asia and the Americas [13]. *Symplocos sumuntia* Buch.-Ham. ex D. Don (*S*. *sumuntia*) is a traditional medicinal herb in Asia [14]. Recently, herbal plants have gained considerable awareness and interest in the field of natural medicine owing to fewer side effects than allopathy [15]. The roots, leaves, and flowers of *S*. *sumuntia* have been used in traditional medicine to treat inflammation, cough, tonsillitis, stomachache, hyperlipidemia, and hypertension [15]. In addition, it has been reported that the leaves of *S*. *sumuntia* inhibit iNOS and cyclooxygenase-2 (COX-2) expression in lipopolysaccharide (LPS)-stimulated RAW 264.7 cells, as well as the subsequent production of NO, a proinflammatory mediator [16]. Although the anti-inflammatory activity of *S. sumuntia* has been reported, little is known about its anti-inflammatory activity and molecular mechanisms in microglial cells.

Therefore, we investigated the molecular mechanisms underlying the anti-inflammatory effects of *S*. *sumuntia* methanol extracts (SSME) on LPS-stimulated BV2 murine microglial cells. This study investigated whether the anti-inflammatory activity of SSME occurs via the inhibition of NO production and the expression of iNOS and COX-2 proteins.

## 2. Results

### 2.1. Analysis of Active Components in SSME

**Figure 1:**
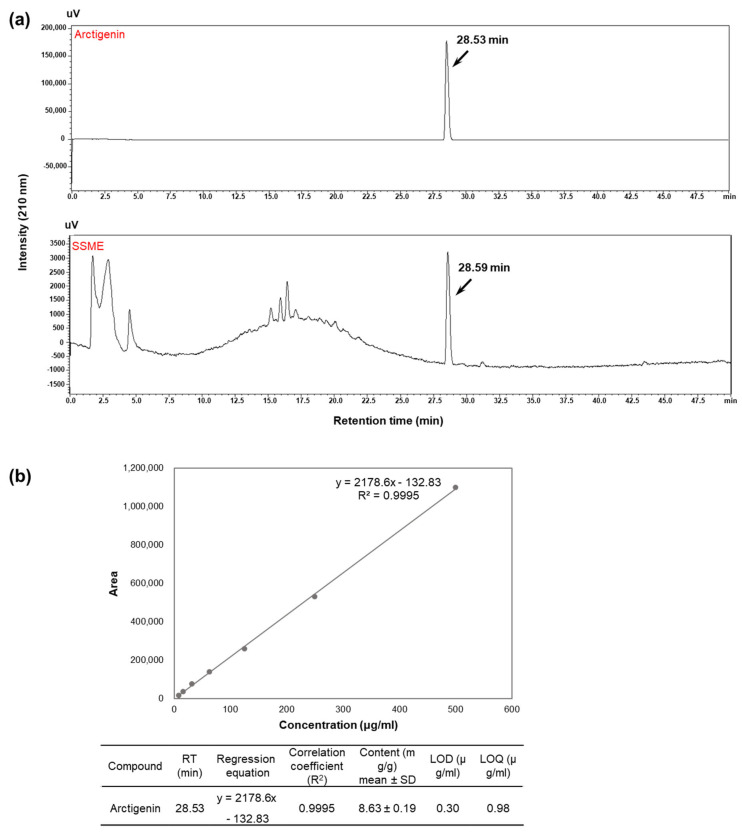
Identification of active compounds in the methanol extract of *Symplocos sumuntia* Buch.-Ham. ex D. Don (SSBE) and its quantitative analysis. (**a**) The phytochemical characteristics of SSBE and arctigenin were detected using HPLC analysis. (**b**) The calibration curve of arctigenin was drawn with concentrations ranging from 500 to 7.81 μg/mL. The quantitative result was expressed as mg of compounds per 1 g of SSME.

Before investigating the effects of SSME on inflammation, an evaluation of the major components that exhibit anti-inflammatory effects was performed. A previous study revealed four lignans, arctigenin, matairesinol, monomethylpinoresinol, and pinoresinol, as the major components of SSME through a phytochemical assay-guided fractionation. All four compounds inhibited NO production, with arctigenin showing the most potent activity with a half-maximal inhibitory concentration (IC_50_) value [16]. Thus, the SSME was analyzed via qualitative HPLC-UV and compared to authentic standards of arctigenin, which showed the presence of a prominent peak for the SSME with the same retention time as arctigenin (Figure 1). Based on the quantitative HPLC analysis and NO assay, it was revealed that the IC_50_ of the arctigenin in the BV2 cells was 2.29 ± 0.58 μg/mL, and the arctigenin content in SSME IC_50_ in the BV2 cells was 1.184 ± 0.02 μg/mL. This indicates the inhibitory effect of the SSME on the NO production was mainly dependent on the arctigenin; however, it was not solely dependent on the effect of the arctigenin. These results suggest that arctigenin as a constituent, which is likely relevant for the anti-inflammatory effects observed for the SSME, showed the advantage of an extract containing multiple active constituents over arctigenin alone and led us to study the anti-inflammatory effects and underlying regulatory mechanism of the action of SSME in BV2 cells.

### 2.2. SSME Exerted No Significant Cytotoxicity on BV2 Cells

**Figure 2:**
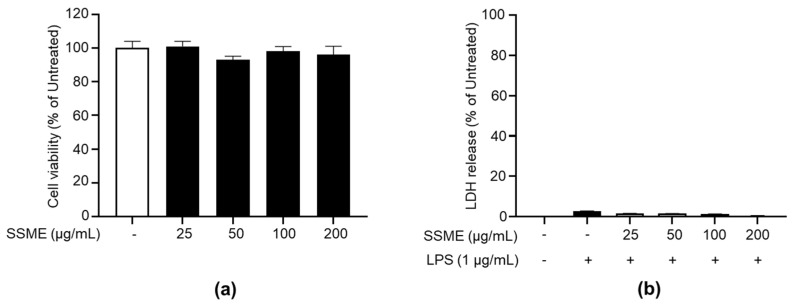
Effect of the SSME on the viability of the BV2 cells. (**a**) The BV2 cells were treated with/without SSME (25, 50, 100, and 200 µg/mL) for 24 h. Cell viability was measured using the EZ-Cytox kit. (**b**) The BV2 cells were treated with SSME (50, 100, and 200 µg/mL) for 2 h, followed by stimulation with LPS (1 µg/mL) for 24 h. The SSME cytotoxicity was measured using the LDH assay kit. The data presented are the mean ± SEM of three independent experiments. Differences between groups were analyzed using the Mann–Whitney U test. SSME, *Symplocos sumuntia* Buch.-Ham. ex D. Don. methanol extract; LPS, lipopolysaccharide; LDH, lactate dehydrogenase.

Cytotoxicity was evaluated in BV2 cells cultured in the presence or absence of SSME (25–200 µg/mL). The EZ-Cytox reagent assay showed no significant difference with or without SSME treatment (Figure 2a). SSME-treated BV2 cells did not show significant changes in cell death with or without LPS treatment. We then evaluated the effect of the SSME (25–200 µg/mL) on the lactose dehydrogenase (LDH) release by LPS-treated BV2 cells. We found that the SSME did not induce significant LDH release in the BV2 cells (Figure 2b). Collectively, the subsequent experiments were performed with the SSME at concentrations of 25–200 µg/mL, which were shown not to exert cytotoxic effects in the BV2 cells.

### 2.3. SSME Inhibited NO Production in LPS-Stimulated BV2 Cells

**Figure 3:**
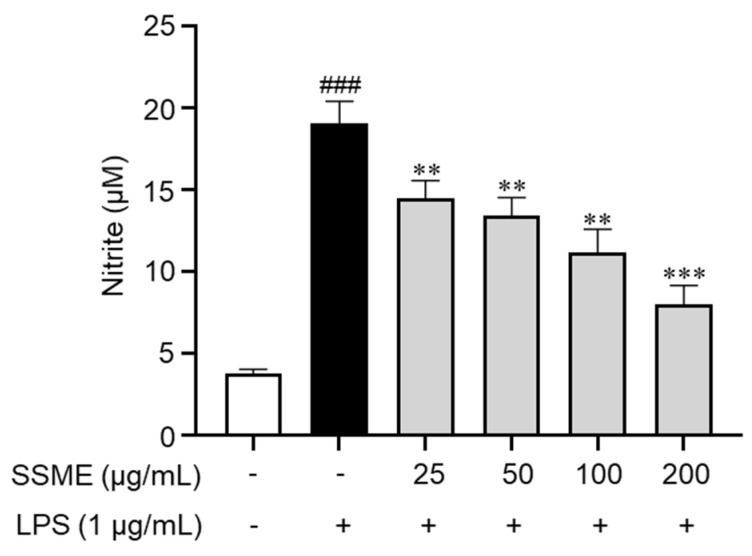
Effect of the SSME on the intracellular NO production in the BV2 cells. The BV2 cells were treated with SSME (50, 100, and 200 µg/mL) for 2 h, followed by stimulation with LPS (1 µg/mL) for 24 h. Intracellular NO secretion was measured using the Griess assay. The data are presented as the mean ± SEM of three independent experiments. Differences between the groups were analyzed using the Mann–Whitney U test. ### *p* < 0.001 compared with the untreated group and the LPS-treated groups; ** *p* < 0.01, *** *p* < 0.001 compared with the LPS-treated and SSME- and LPS-treated groups. SSME: *Symplocos sumuntia* Buch.-Ham. ex D. Don. methanol extract; LPS: lipopolysaccharide; NO: nitric oxide.

To investigate the anti-inflammatory effects of the SSME, we evaluated intracellular NO production, a well-known proinflammatory mediator in BV2 cells. Subsequent experiments were performed at SSME concentrations of 25, 50, 100, and 200 µg/mL, which, according to our results, did not exert cytotoxic effects. Nitrite production increased in the LPS-stimulated BV2 cells. However, in the cells pretreated with SSME, LPS-induced NO production was dose-dependently suppressed (Figure 3). These results suggest that the SSME markedly reduced NO production in the LPS-stimulated BV2 cells.

### 2.4. SSME Suppressed iNOS and COX-2 Expression in LPS-Activated BV2 Cells

**Figure 4:**
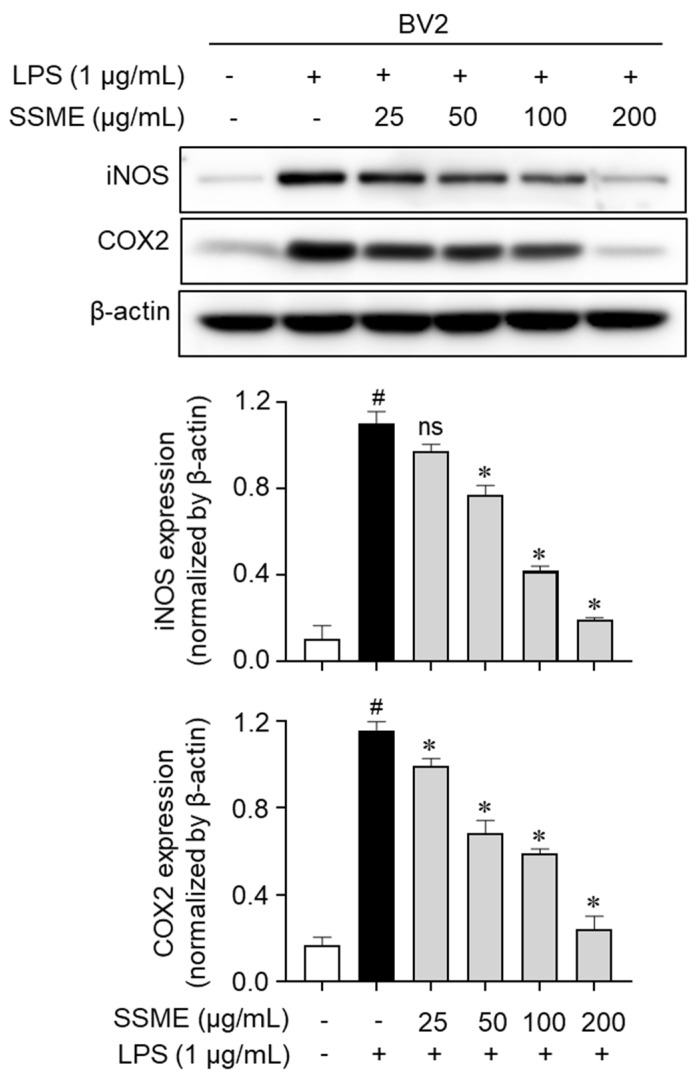
Effects of the SSME on the iNOS and COX-2 expression in the BV2 cells. The BV2 cells were treated with SSME (50, 100, and 200 µg/mL) for 2 h, followed by stimulation with LPS (1 µg/mL) for 24 h. After stimulation, the iNOS and COX-2 protein levels were analyzed by western blotting. The data are presented as the mean ± SEM of three independent experiments. Differences between the groups were analyzed using the Mann–Whitney U test. # *p* < 0.05, compared with the untreated and the LPS-treated groups; * *p* < 0.05, compared with the LPS-treated and SSME- and LPS-treated groups; ns, not significant. iNOS, inducible nitric oxide synthase; COX-2: cyclooxygenase-2, SSME: *Symplocos sumuntia* Buch.-Ham. ex D. Don. methanol extract; LPS: lipopolysaccharide.

The proinflammatory enzymes, including COX-2 and iNOS, play important roles in the immune response of activated microglial cells through NO and PGE_2_ production [10]. We investigated the effect of the SSME on the expression of iNOS and COX-2 in the LPS-stimulated BV2 cells using western blotting. The iNOS and COX-2 expression was significantly increased in the LPS-stimulated BV2 cells (Figure 4). However, the BV2 cells treated with the SSME showed a dose-dependent decrease in iNOS and COX-2 expression (Figure 4). These results indicate that the SSME suppressed the proinflammatory mediator production by inhibiting the expression of the proinflammatory enzymes iNOS and COX-2.

### 2.5. SSME Regulated Proinflammatory Cytokine Production in LPS-Induced BV2 Cells

**Figure 5:**
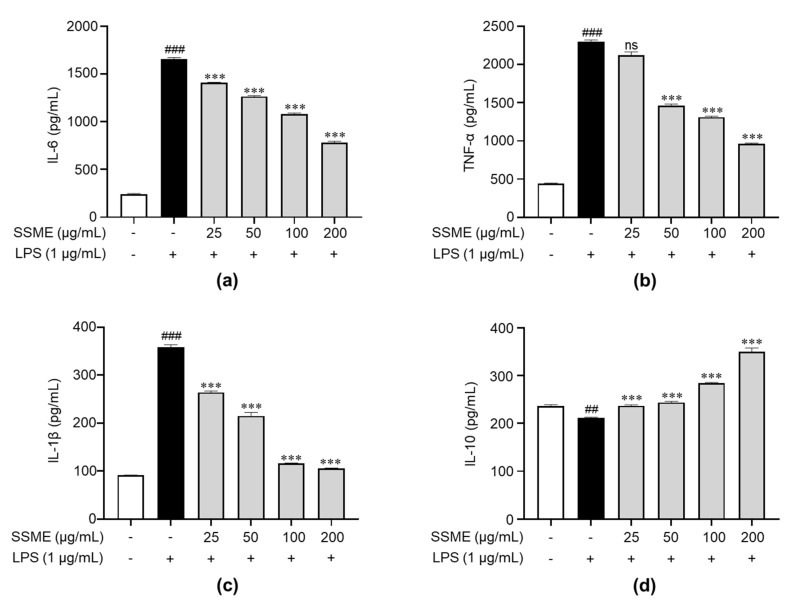
Effects of the SSME on proinflammatory cytokine production in the BV2 cells. The BV2 cells were treated with SSME (50, 100, and 200 µg/mL) for 2 h, followed by stimulation with LPS (1 µg/mL) for 24 h. SSME (**a**–**d**) After LPS activation for 24 h, the production of IL-6, TNF-α, IL-1β, and IL-10 was detected using ELISA. The data are presented as the mean ± SEM of three independent experiments. Differences between the groups were analyzed using the Mann–Whitney *U* test. ## *p* < 0.01, ### *p* < 0.001 compared with the untreated group and the LPS-treated groups; *** *p* < 0.001 compared with the LPS- and SSME- and LPS-treated groups; ns, not significant. IL, interleukin; TNF, tumor necrosis factor.

To investigate whether the SSME affected the production of proinflammatory and anti-inflammatory cytokines, we evaluated the effect of the SSME on the production of IL-1β, IL-6, and TNF-α (proinflammatory cytokines) and IL-10 (anti-inflammatory cytokine) in the LPS-stimulated BV2 cells using ELISA. The LPS treatment markedly induced the production of proinflammatory cytokines, such as TNF-α, IL-6, and IL-1β, but dose-dependently suppressed the production after pretreatment with SSME in the BV2 cells (Figure 5a–c). Additionally, LPS treatment markedly reduced the production of anti-inflammatory cytokines such as IL-10 in the BV2 cells. However, the BV2 cells pretreated with the SSME showed a dose-dependent increase in IL-10 production (Figure 5d). These results suggest that the SSME induces anti-inflammatory effects by inhibiting proinflammatory cytokines, such as IL-6, TNF-α, and IL-1β, and inducing anti-inflammatory cytokines, such as IL-10.

### 2.6. SSME Suppressed JNK Phosphorylation in LPS-Stimulated BV2 Cells

**Figure 6:**
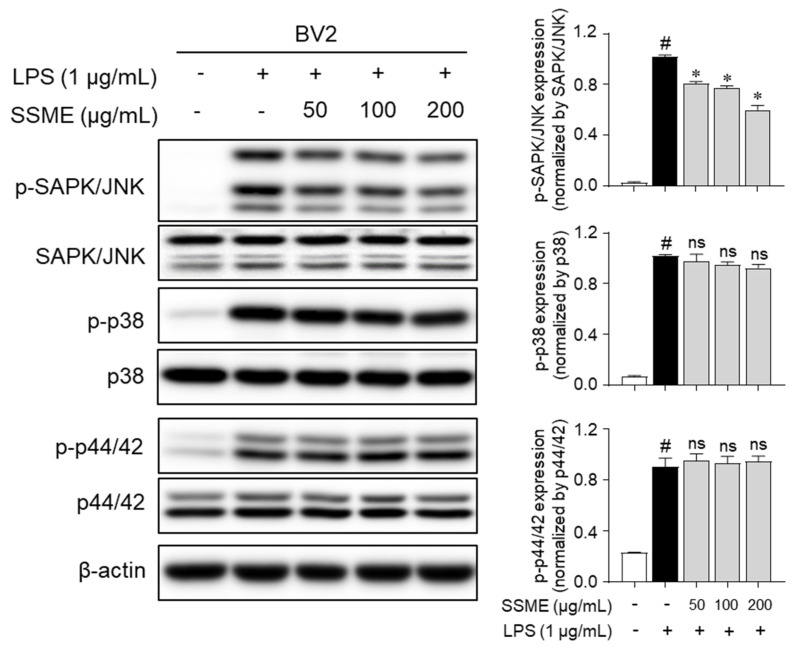
Effects of the SSME on the MAPK signaling pathway. The BV2 cells were treated with SSME (50, 100, and 200 µg/mL) for 2 h, followed by stimulation with LPS (1 µg/mL) for 15 min. The cellular protein levels of p38, p44/42 (Erk1/2), and JNK were measured by western blotting. The data are presented as the mean ± SEM of three independent experiments. Differences between the groups were analyzed using the Mann–Whitney U test. # *p* < 0.05, compared with the untreated group and the LPS-treated groups; * *p* < 0.05, compared with the LPS-treated and SSME- and LPS-treated groups. p-, phosphorylated; SAPK/JNK, stress-associated protein kinase/c-Jun N-terminal kinase; ns, not significant.

MAPK signaling pathways are major regulators of the expression of inflammatory mediators [17,18]. To elucidate the mechanisms underlying the anti-inflammatory effects of the SSME, we investigated whether the SSME regulated the LPS-induced phosphorylation of MAPKs, including JNK, p38, and p44/42, using western blotting. Figure 6 shows that the LPS-induced phosphorylation of JNK was significantly suppressed in a dose-dependent manner after pretreatment with SSME in the BV2 cells. Pretreatment with SSME did not suppress the LPS-induced phosphorylation of p38 or p44/42 in the BV2 cells (Figure 6). These results suggest that the anti-inflammatory effects of the SSME were mediated by the suppression of JNK signaling but not p38 and p44/42.

### 2.7. SSME Suppressed NF-κB Activation in LPS-Stimulated BV2 Cells

**Figure 7:**
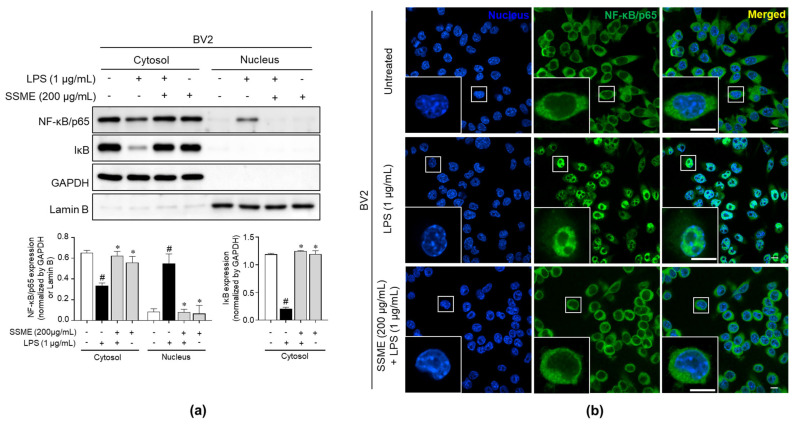
Inhibitory effects of the SSME on the NF-κB signaling pathway. The BV2 cells were treated with SSME (200 µg/mL) for 2 h, followed by stimulation with LPS (1 µg/mL) for 15 min. (**a**) The expression of NF-κB/p65 and IκB was detected in the cytosolic and nuclear extracts using western blotting. (**b**) The cells were immunostained with anti-NF-κB/p65 (green) antibody, and nuclei were stained with DAPI (blue). The cells were analyzed with confocal imaging microscopy. The data presented are the mean ± SEM of three independent experiments. Differences between groups were analyzed using the Mann–Whitney U test. # *p* < compared with the untreated and the LPS-treated groups; * *p* < 0.05, compared with the LPS-treated and SSME- and LPS-treated groups. NF-κB, nuclear factor kappa-light-chain-enhancer of activated B cells; IκB, inhibitor of κB; DAPI, 4′,6-diamidino-2-phenylindole. Scale bar: 10 μm.

NF-κB, a pivotal mediator of inflammatory responses, regulates innate and adaptive immune functions [19]. The translocation of NF-κB from the cytoplasm to the nucleus is a critical step in the coupling of extracellular stimuli to the transcriptional activation of specific target genes [19]. To elucidate the mechanisms underlying the anti-inflammatory effects of the SSME, we examined the changes in the NF-κB translocation into the nucleus after treatment with SSME. Figure 7a shows that the LPS-stimulated degradation of IκB was significantly inhibited after pretreatment with SSME in the cytosolic fraction. In contrast, the level of the LPS-induced nuclear NF-κB/p65 protein, which translocates into the nucleus after IκB degradation, was decreased by the SSME pretreatment in the BV2 cells (Figure 7a). We also confirmed the translocation of the NF-κB/p65 protein with/without SSME treatment in the BV2 cells by immunofluorescence staining. The SSME-treated BV2 cells showed reduced translocation of NF-κB/p65 from the cytosol to the nucleus, which increased trafficking into the nucleus following LPS stimulation (Figure 7b). These results suggest that the SSME induced anti-inflammatory effects via inhibition of the activation of the NF-κB signaling pathway in the BV2 cells.

## 3. Discussion

Recently, plants have become important sources of drugs that target various diseases, including inflammatory disorders [20,21,22,23]. Many researchers have reported significant anti-inflammatory effects for plant extracts and formulations thereof [24,25,26,27,28,29]. Huong et al. [16] reported that the *S. sumuntia* extract induced anti-inflammatory activity via the inhibition of NO production in LPS-stimulated RAW264.7 cells. However, little is known about the molecular mechanism underlying its anti-inflammatory effects in microglial cells. Microglia-mediated inflammatory responses play an important role in Parkinson’s, Alzheimer’s, and other cerebral diseases [5]. In this study, we investigated the anti-inflammatory effects and mechanism of action of the SSME in LPS-stimulated BV2 microglial cells.

In neurodegenerative diseases, microglial cells and astrocytes are the major cells involved in the CNS immune responses. Microglial cells, a type of macrophage, are responsible for the innate immune response to inflammatory responses in the central nervous system [5]. Hyperactive cells also lead to disastrous and progressive neuropathological damage due to the excessive production of a large array of cytotoxic mediators, including superoxide, NO, and proinflammatory cytokines [TNF-α, IL-1β, IL-6, and monocyte chemoattractant protein-1 (MCP-1)] [30]. Chronic production of these cytokines is directly involved in the pathogenesis of several neuroinflammatory and neurodegenerative diseases, such as neuromyelitis optical spectrum disorder and cerebral interferonopathy, respectively [30]. Understanding the regulation of microglial activation is critical to comprehending the inflammatory process in CNS pathology and provides ideal prospects for targeted anti-inflammatory therapy capable of slowing and preventing the progression of neuroinflammatory diseases [31,32]. Therefore, the inhibitory effects of plant extracts and those isolated compounds via the suppression of proinflammatory cytokines have been suggested for treating inflammatory diseases. Compounds isolated from *Cullen corylifolium* (L.) Medik. extract were reported to decrease LPS-induced oxidative stress and the expression of inflammatory cytokines and to provide a neuroprotective effect by antagonizing microglia-mediated inflammation for attenuated PD [33]. In this study, we evaluated the inhibitory effects of SSME on inflammatory responses in LPS-stimulated macrophages and found that the SSME significantly regulated the levels of pro- and anti-inflammatory cytokines, such as IL-6, TNF-α, IL-1β, and IL-10, in the BV2 cells (Figure 3 and Figure 4). These results suggest that SSME exerts potent anti-inflammatory effects by regulating NO/iNOS production and pro- and anti-inflammatory cytokine levels.

It has been reported that NF-κB and MAPKs signaling pathways are the major downstream pathways of TLR4 in the inflammatory response [34]. Binding complexes of IκB and NF-κB cannot translocate to the nucleus from the cytoplasm, and their activation is also limited [35]. However, NF-κB can be released via IκB phosphorylation during the LPS-stimulated inflammatory response [35]. Subsequently, the p65 subunit separated from the NF-κB complex translocates from the cytoplasm to the nucleus and triggers the transcription of target genes and cytokines, such as IL-6, TNF-α, IL-1β, iNOS, and COX-2, through the canonical NF-κB pathway in the immune response [36,37,38,39]. The MAPK signaling pathway is pivotal in regulating the inflammatory process [17]. The mammalian MAPK family consists of JNK, p38, and p44/42, which phosphorylate other protein kinases and are involved in gene transcription and inflammation [17,18,40,41]. Our study showed that JNK phosphorylation in LPS stimulation was suppressed by the SSME in the BV2 cells (Figure 6). Moreover, the SSME inhibited the NF-κB pathway by inhibiting the LPS-induced transfer of the NF-κB subunit from the cytoplasm to the nucleus in the BV2 cells (Figure 7).

In conclusion, this study showed that SSME exerted anti-inflammatory effects by inhibiting oxidative stress in the LPS-induced BV2 microglial cells. Our findings reveal the potential molecular mechanism of SSME in anti-inflammatory responses, which is involved in the regulation of iNOS, COX-2, proinflammatory cytokines (IL-6, TNF-α, and IL-1β), anti-inflammatory cytokine (IL-10), and NO production via blockade of the NF-κB/p65 and JNK pathways in the LPS-stimulated BV2 cells. Collectively, these results suggest that SSME may be a potential plant extract for use in neurodegenerative diseases.

## 4. Materials and Methods

### 4.1. Plant Extract

*S. sumuntia* was collected from the Da Chais community, Lac Duong district, Lam Dong province, Vietnam. Plant samples were collected and identified by Dr. Tran The Bach at the Institute of Ecology and Biological Resources (Hanoi, Vietnam). Voucher specimens were recorded as KRIB41299 and VK4863 and have been deposited at the herbarium of the Republic of Korea Research Institute of Bioscience and Biotechnology (Daejeon, Republic of Korea). The plant extract was prepared as described below. Briefly, the plant (90 g) was dried in the shade, powdered, added to 1 L of Methanol (HPLC Grade), and extracted through 30 cycles (40 KHz, 1500 W, 15 min. ultrasonication-120 min. standing per cycle) at room temperature using an ultrasonic extractor (SDN-900H, SD-ULTRASONIC Co., Ltd.). After filtration and drying under reduced pressure, the *S. sumuntia* extract (6.0 g) was obtained. The yield of the *S. sumuntia* methanol extract was 6.45%.

### 4.2. Quantitative HPLC Analysis

The quantification of arctigenin was analyzed using an HPLC system (Shimadzu Corp., Kyoto, Japan) with an SPD-20A UV/Vis detector, an LC-20AR solvent pump. Chromatography was achieved on an Atlantis T3 Column (4.6 mm × 250 mm, 5 μm) and monitored at 210 nm. The arctigenin standard compound was dissolved in 100% MeOH to the concentration of 1 mg/mL and diluted with MeOH. Linear gradients at a flow rate of 1 mL/min with H_2_O (A) and CH_3_CN (B) were applied as follows: 0–40 min, 10 to 90% B, 40–50 min, 90% to 100% B, and the injection volume was 10 μL. The calibration curve of the arctigenin was drawn with concentrations ranging from 500 to 7.81 μg/mL. The quantitative determination of the sample was carried out in triplicate. The quantitative result was expressed as mg of compounds per 1 g of SSME. The regression equation was calculated as y = ax + b, where y and x corresponded to the peak area and concentration, respectively. The linearity was established through the linear correlation coefficient (R^2^) of the calibration curve for arctigenin. The limit of detection (LOD) and limit of quantification (LOQ) were calculated by injecting a series of diluted standard solutions at signal to noise ratios (S/N) of almost 3 and 10, respectively.

### 4.3. Cell Culture

BV2 murine microglial cells were purchased from AcceGen (Fairfield, NJ, USA). The BV2 cells were cultured in Dulbecco’s modified Eagle’s medium (DMEM, #LM0001-05; WELGENE Inc., Gyeongsan, Republic of Korea) containing 1% penicillin-streptomycin (#30-002-CI; Corning Inc., Corning, NY, USA) and 10% fetal bovine serum (FBS) (#16000044; Thermo Fisher Scientific Inc., Waltham, MA, USA) in an incubator at 37 °C under 5% CO_2_ conditions.

### 4.4. Cell Viability Assay

The cell viability assay was performed using an EZ-Cytox kit (#EZ-1000; DoGen Bio, Seoul, Republic of Korea), according to the manufacturer’s protocol. Briefly, the cells were seeded on a 96-well plate in triplicates and treated with the SSME (25, 50, 100, and 200 µg/mL) for 24 h in an incubator at 37 °C with 5% CO_2_. Then, the cells were incubated with EZ-Cytox reagent in a CO_2_ incubator at 37 °C for 30 min. The optical density (OD) of the samples was measured at 450 nm using a microplate reader (Molecular Devices, San Jose, CA, USA). The control cells (untreated) contained an equivalent amount of conditioned medium added to the wells.

### 4.5. Cytotoxicity Assay

Cytotoxicity was determined by the release of lactate dehydrogenase (LDH) from dead cells using the EZ-LDH kit (#DG-LDH500; DoGen Bio), according to the manufacturer’s protocol. The cells were seeded in triplicate and pretreated with SSME (25, 50, 100, and 200 µg/mL) for 2 h. After pretreatment, the cells were stimulated with LPS from *Escherichia coli* O127:B8 (1 µg/mL, #L4516; Sigma-Aldrich, St. Louis, MO, USA) for 24 h. The cell culture supernatants were then collected and mixed with EZ-LDH reagent at room temperature (RT) in the dark for 30 min. Finally, the OD of the samples was measured at 450 nm using a microplate reader. Cytotoxicity was calculated following [42]. The following conditions were used as controls to calculate the cytotoxicity: (1) A background control was used to measure the OD of the LDH from the complete medium. (2) A high control group with lysing solution-treated cells was used to determine the maximum amount of LDH released from the lysed cells. (3) There was a volume control group where lysis solution was added to the completed medium. (4) There was a low control used to determine the minimum amount of LDH released by cells that died naturally. Cytotoxicity was calculated using the following equation: Cytotoxicity (%) = [(OD_450_ experimental − OD_450_ background control) − (OD_450_ low control − OD_450_ background control)]/[(OD_450_ high control − OD_450_ volume control) − (OD_450_ low control − OD_450_ background control)] × 100.

### 4.6. Nitric Oxide Assay

The cells were pretreated with the SSME (25, 50, 100, and 200 µg/mL) for 2 h and stimulated with LPS (1 µg/mL) for 24 h. Then, the cell culture supernatants were collected and mixed with Griess reagent [1% sulfanilamide (#S9251), 0.1% N-1-naphthylenediamine dihydrochloride (#N9125; Sigma-Aldrich, St. Louis, MO, USA), and 2.5% phosphoric acid (#1.00573.1000; Merck Millipore, Burlington, MA, USA)] at RT for 5 min. Finally, the absorbance of the samples was measured at a wavelength of 540 nm using a microplate reader.

### 4.7. Western Blot Analysis

The cells were pretreated with the SSME (25, 50, 100, and 200 µg/mL) for 2 h and stimulated with LPS (1 µg/mL) for 15 min or 24 h. The cells were lysed with radioimmunoprecipitation assay (RIPA) buffer (#RC2002-050-00; Biosesang, Seongnam, Republic of Korea) containing protease inhibitor cocktail (#P8340) and phosphatase inhibitor cocktail II (#P5726) and III (#P0044; Sigma-Aldrich). The cell lysates were sonicated (Sonics & Materials Inc., Newtown, CT, USA) and denatured. The prepared samples were subjected to sodium dodecyl sulfate–polyacrylamide gel electrophoresis and transferred to nitrocellulose membranes. The membranes were blocked in phosphate-buffered saline (PBS)-Tween 20 (#TW2001; LPS Solution, Daejeon, Republic of Korea) containing 5% skim milk (#SKI500; LPS Solution). The membranes were incubated with anti-iNOS (1:1000; #610332; BD Biosciences, San Diego, CA, USA), anti-COX2 (1:1000; #sc-166475), anti-β-actin (1:5000; #sc-47778), anti-p38 (1:2000; #sc-7972), anti-p44/42 (1:2000; Erk1/2, #sc-514302) (Santa Cruz Biotechnology Inc., Dallas, TX, USA), anti-SAPK/JNK (1:1000; #9252), anti-phospho-SAPK/JNK (1:1000; #9251), anti-phospho-p38 (1:1000; #9211), and anti-phospho-p44/42 (1:2000; #9101) (Cell Signaling Technology, Danvers, MA, USA) overnight at 4 °C. Each membrane was incubated for 1 h with peroxidase-conjugated anti-mouse (1:2000; #7076) and anti-rabbit (1:2000; #7074) antibodies (Cell Signaling Technology). The protein bands were developed with enhanced chemiluminescence (ECL) solution and quantified using ImageJ software (National Institutes of Health, Bethesda, MD, USA).

### 4.8. Enzyme-Linked Immunosorbent Assay (ELISA)

The cells were pretreated with SSME (25, 50, 100, and 200 µg/mL) for 2 h and stimulated with LPS (1 µg/mL) for 24 h. After stimulation, the levels of each cytokine were measured in the cell culture supernatants. The purified anti-IL-6 (1:250; #554400), anti-IL-10 (1:250; #551215) (BD Bioscience), anti-IL-1β (1:200; #14-7012-85), and anti-TNF-α (1:250; #14-7423-85) (Thermo Fisher Scientific Inc.) antibodies were coated onto 96-well plates and incubated overnight at 4 °C. Next, the plates were washed with PBS containing 0.05% Tween 20 and incubated with PBS containing 1% bovine serum albumin (BSA) at RT for 1 h. After that, the plates were washed, and the supernatant samples were added for 2 h. The plates were washed, and the detection antibodies were added for 1 h. Next, streptavidin-conjugated alkaline phosphatase (1:250; AKP, #554065; BD Bioscience) was added after washing. Substrate buffer [10% diethanolamine (#3032-4400), 0.1% MgCl_2_·6H_2_O (#5503-44), 0.2% NaN_3_ (#7530-4105) (Daejung Chemicals & metals Co., Siheung, Republic of Korea) and 4-nitrophenyl phosphate (#N2765, Sigma-Aldrich)] was added to each well. The absorbance of the samples was measured at 405 nm using a microplate reader.

### 4.9. Nucleus/Cytoplasm Fractionation

Fractionation was performed using the NE-PER Nuclear and Cytoplasmic Extraction Reagent Kit (#78833; Thermo Fisher Scientific Inc.), according to the manufacturer’s protocol. The cells were pretreated with SSME (25, 50, 100, and 200 µg/mL) for 2 h and stimulated with LPS (1 µg/mL) for 15 min. After incubation, the cells were harvested and centrifuged 500× *g* for 5 min at 4 °C, and the supernatants were carefully discarded. Next, the cells were vortexed with lysis solution, and the supernatants (cytoplasmic extract) were harvested through centrifugation. The pellets were then vortexed with nuclear lysis solution and centrifuged at the maximum speed. The supernatants (nuclear extract) were then transferred to new pre-chilled tubes. Finally, the supernatants were denatured and subjected to western blot analysis. Lamin B1 (1:700; nuclear marker; #sc-377000), GAPDH (1:1000; cytosolic marker; #sc-365062), NF-κB p65 (1:700; #sc-8008; Santa Cruz Biotechnology Inc.), and IκB (1:1000; #9242; Cell Signaling Technology) were used to detect the protein bands.

### 4.10. Immunofluorescence

The cells were pretreated with SSME (25, 50, 100, and 200 µg/mL) for 2 h and stimulated with LPS (1 µg/mL) for 15 min. The cells were washed with PBS and fixed using 4% paraformaldehyde solution (#PC2031-050-00; Biosesang) at RT for 10 min. After fixation, the cells were washed with PBS and permeabilized with 0.1% Triton X-100 (#T8787; Sigma-Aldrich) for 10 min at RT. The cells were incubated with PBS containing 1% BSA at RT for 1 h. After washing, the cells were incubated with an NF-κB antibody (diluted at 1:100) at 4 °C overnight. The cells were then incubated with the secondary antibodies in the dark for 1 h. The cells were fixed onto glass slides using a mounting solution (#S36936; Thermo Fisher Scientific Inc.), and fluorescent images were captured using a confocal microscope (Model; Nikon AX R, Nikon Instruments, Tokyo, Japan).

### 4.11. Statistical Analysis

All experiments were performed in triplicate. The data are expressed as the mean ± standard error of the mean and were analyzed using GraphPad Prism software (version 8.0 GraphPad Inc., San Diego, CA, USA). The results were analyzed using the nonparametric Mann–Whitney U test, with a value of *p* < 0.05 considered to indicate a significant difference.

## Data Availability

Not applicable.
